# Follow-up outcomes of asymptomatic brucellosis: a systematic review and meta-analysis

**DOI:** 10.1080/22221751.2023.2185464

**Published:** 2023-03-13

**Authors:** Fande Li, Lanping Du, Hua Zhen, Mujinyan Li, Shuqi An, Wenqi Fan, Yuke Yan, Meifang Zhao, Xin Han, Zhuo Li, Huixin Yang, Cui Zhang, Chao Guo, Qing Zhen

**Affiliations:** a Department of Epidemiology and Biostatistics, Key Laboratory of Zoonosis, Ministry of Education, School of Public Health, Jilin University, Changchun, People’s Republic of China; b Beijing Tiantan Hospital, Capital Medical University, Beijing, People’s Republic of China.

**Keywords:** Brucellosis, asymptomatic, follow-up outcomes, prevalence, public health, meta-analysis

## Abstract

Balancing the potentially serious outcomes of asymptomatic brucellosis and “waiting” for treatment in clinical practice is an urgent issue. Therefore, we assessed the follow-up outcomes and epidemiological characteristics of asymptomatic brucellosis in the absence of treatment to provide evidence-based clinical clues. We searched eight databases in which 3610 studies from 1990 to 2021 were related to the follow-up outcomes of asymptomatic brucellosis. Thirteen studies, involving 107 cases, were finally included. Regarding the follow-up outcomes, we examined the presence or absence of symptoms and decreased serum agglutination test (SAT) titre. During the 0.5–18 months follow-up period, the pooled prevalence of appearing symptomatic was 15.4% (95% CI 2.1%–34.3%), cases that remained asymptomatic were 40.3% (95% CI 16.6%–65.8%), and decreased SAT titre was observed in 36.5% (95% CI 11.6%–66.1%). Subgroup analysis indicated that the pooled prevalence of appearing symptomatic with follow-up times of less than 6 months, 6–12 months, and 12–18 months was 11.5%, 26.4%, and 47.6%, respectively. The student subgroup had a higher prevalence of symptoms (46.6%) than the occupational and family populations. In conclusion, asymptomatic brucellosis has a high likelihood of appearing symptomatic and its severity may be underestimated. Active screening of occupational and family populations should be enhanced, and special attention should be paid to high-titre students for early intervention, if necessary. Additionally, future prospective, long-term, and large-sample follow-up studies are essential.

## Introduction

Brucellosis is a highly neglected zoonotic disease caused by *Brucella* spp. and is prevalent worldwide. Humans primarily get infected through the consumption of unpasteurized dairy or meat products, direct contact with infected animals, or laboratory exposure [1]. Although the mortality is low, brucellosis can lead to severe weakness or disability [2, 3].

Asymptomatic brucellosis is a specific type of brucellosis that is seropositive without any brucellosis-related symptoms. Its cases were observed in the range of 16.3% to 92.0% during the active screening of high-risk populations [4–9]. However, concerning that overdiagnosis and treatment may result in untoward drug effects [10], there are few diagnoses and interventions for asymptomatic infection in clinical practice. Less than 10% of occupationally exposed asymptomatic individuals are diagnosed with the infection at an early stage [1,11]. Besides the use of prophylactic medication for laboratory personnel at high risk from *Brucella* leakage and clinical intervention for some high antibody titre infections (especially in pregnant women and children) after comprehensive physician evaluation, most asymptomatic brucellosis cases are overlooked [12–15].

For asymptomatic individuals, if the onset process is not controlled effectively, there may be a risk of serious physical damage as well as a potential risk of disease transmission. Low immunity, for example, human immunodeficiency virus infection, leukaemia, and organ transplantation, may lead to clinical changes from asymptomatic to flare [16,17]. If not treated in the early stages, it can easily to turn into chronic, with the potential for serious complications, such as spondylitis, endocarditis, and adverse pregnancy outcomes [18–20]. Additionally, some asymptomatic individuals have been isolated *brucella* [21,22], which can be transmitted through special routes such as organ transplantation, blood transfusion, sexual activity, or breast milk [23–26].

Determining the outcomes of asymptomatic brucellosis is an urgent problem as we have to deal with epidemics of brucellosis everywhere and we also have to confront the challenges posed by major public health events at a given time. In 2019, The Lanzhou *Brucella* Leak Event (the largest laboratory accident in the history of infectious diseases) took this issue to a new level [27]. The accident was caused by exhaust emissions of the *brucella* vaccine, which was not completely disinfected because of outdated disinfectants. As of November 30, 2020, 10,528 (15.4%) residents had positive serum antibody titres and most of them were asymptomatic [28]. The conflict between the patient’s concerns about the serious consequences of asymptomatic brucellosis and the hospital’s failure to diagnose and treat the disease according to the guidelines has caused many difficulties in subsequent management and compensation. Fundamentally, this is caused by the uncertainty in the outcomes of asymptomatic brucellosis.

To our knowledge, no systematic review of outcomes in asymptomatic patients with brucellosis has been published, although research on brucellosis has intensified in recent years. There are few studies available on the outcomes of asymptomatic brucellosis and most of them are case reports. Some studies reported high rates of appearing symptomatic [5,29], while others reported high rates of asymptomatic disease [30] or even decreased serum agglutination test (SAT) titre [31,32]. Therefore, we assessed the pooled prevalence of follow-up outcomes for asymptomatic brucellosis and explored related epidemic characteristics. This will help to better determine the propensity for outcomes in asymptomatic brucellosis and provide evidence-based conclusions for the clinical management and risk assessment of asymptomatic brucellosis.

## Methods

### Literature search

This study was conducted according to PRISMA (Preferred Reporting Items for Systematic Reviews and Meta-Analyses) guidelines and registered with PROSPERO (CRD42021254167). We searched all English and Chinese literature on the follow-up outcomes of asymptomatic brucellosis patients published between January 1990 and December 2021. These included four English databases (PubMed, Web of Science, Scopus, and Embase) and four Chinese databases (China National Knowledge Infrastructure (CNKI), WanFang databases, VIP Chinese Science and Technology Periodicals Database (VIP), and SinoMed). Search was performed using the following keywords: “Brucellosis,” “Malta Fever,” “Rock Fever,” “Asymptomatic,” “Subclinical,” “Inapparent,” “Presymptomatic,” “Family,” “Occupation,” “Follow-up study” and “Outcome.” Based on different databases, the search strategies were adjusted to include combinations of MeSH and free words.

The search strategies used for all the databases are presented in S1 appendix. We also searched Google Scholar and the reference lists of relevant studies to locate additional studies that might have been missed during the search performed using the aforementioned electronic databases.

### Follow-up outcomes

Based on existing studies and clinical practice, we defined three main follow-up outcomes: (i) appearing symptomatic: participants had clinical symptoms of brucellosis, such as fever, sweating, fatigue, headache, myalgia, or arthralgia; (ii) maintaining asymptomatic: asymptomatic disease with high SAT titre; (iii) decreased SAT titre: asymptomatic disease with low or negative SAT titre.

### Inclusion and exclusion criteria

Studies were included in the meta-analysis if they met the following criteria: (1) examined participants manifested positive SAT according to respective diagnostic criteria; (2) included patients with no symptoms of brucellosis, such as fever, sweating, malaise, headache, myalgia, and arthritis; and (3) studies reporting the follow-up outcomes and time duration of asymptomatic brucellosis. Studies were excluded if they met the following criteria: (1) full-text version was not available, (2) incomplete data of the follow-up, (3) no asymptomatic participants, (4) participants were diagnosed with brucellosis before, or (5) were treated for brucellosis.

### Literature selection and data extraction

Two authors (FD. L; LP. D) independently screened the articles based on the inclusion and exclusion criteria. Any uncertainties and disagreements were resolved by a third author (H. Z). After identifying the articles for analysis, we extracted data independently using standardized tables. The following information was recorded: first author, year, country, investigation time, age, sex, population (occupation), main animals involved, serological tests, sample size, follow-up time, and outcomes. Detailed data are shown in Table 1.

### Quality assessment

The Agency for Healthcare Research and Quality (AHRQ) [33] performed the quality assessment of the included studies. AHRQ has 11 items including data sources, inclusion criteria, observation time, study population continuity, and quality control. One point was given for “yes,” and zero points for “unclear” or “no.” A total score of 0–3 indicates low-quality, 4–7 medium-quality, and 8–11 high-quality studies. Quality was assessed independently by two authors (FD. L; LP. D) and in the event of disagreement by a third author (X. H). Specific items and the assessment results of the included studies are presented in S2 Appendix.

### Statistical analysis

Meta-analyses were performed using the “meta” package in R 4.0.3. The source data were first transformed by Freeman-Tukey double arcsine or arcsine transformation to make them fit a normal distribution [34], and then a meta-analysis was performed. Heterogeneity among the studies was quantified using *I*^2^ test. Forest plots were used to show pooled prevalence estimates and 95% confidence intervals (CI). If *I*^2 ^> 50%, indicating obvious heterogeneity among the studies, the random effects model was selected to pool the data. If *I*^2 ^≤ 50%, suggesting that data from individual studies had a trend toward homogeneity, the fixed effects model was adopted. Potential publication bias was assessed using funnel plots and Egger’s tests, and *P* values higher than 0.05 indicated that there was no publication bias.

### Subgroup analysis

The sample size and heterogeneity of studies for the two outcomes, maintaining asymptomatic and decreased SAT titre, were relatively small. Hence, only subgroup analyses were conducted for the appearing symptomatic follow-up outcome. Given the characteristics of the studies and disease epidemic, we considered the following subgroups: investigation time (1990–2009 vs. 2010–2021), region (Asia vs. Europe & America), population (occupational population, students, family members), main animals (sheep/goats, cattle, sheep and cattle, camels, sheep, livestock), SAT (≥1:80/≥1:100 vs. ≥1:160/≥1:200), follow-up time (>6 months, 6–12 months, 12–18 months), and quality assessment (high vs. medium). Due to the lack of exact quantitative statistics for age, sex, and history of brucellosis exposure, a subgroup analysis was not performed. The detailed data for the subgroups are shown in S3 Appendix.

## Results

### Study selection and quality assessment

**Figure 1.**
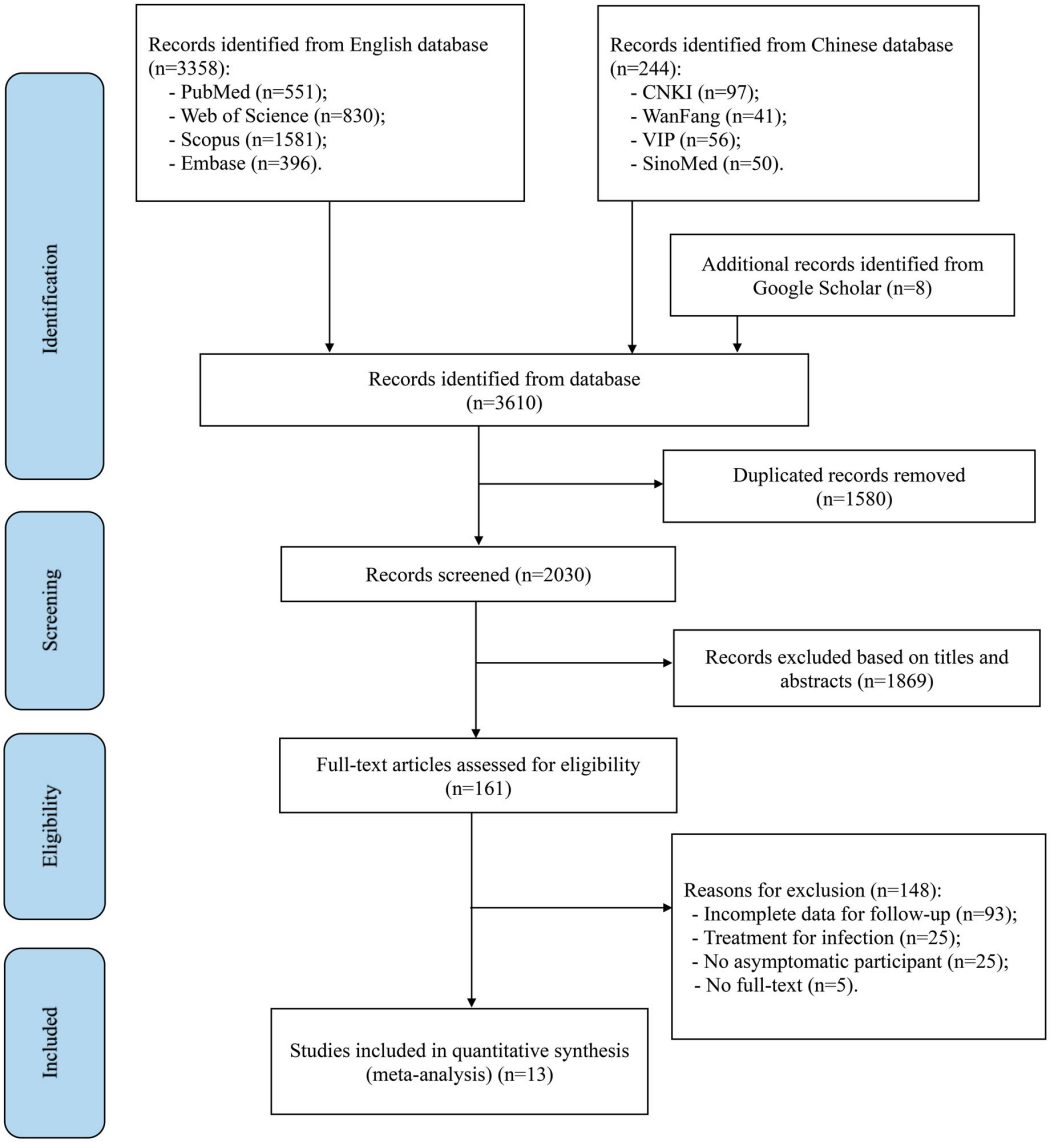
A flow chart of the inclusion criteria of studies eligible for meta-analysis.

A total of 3610 studies were retrieved from eight databases and other resources, including Google Scholar. Of these, 3358 studies were retrieved through English databases (PubMed: 551; Web of SCI: 830; Scopus: 1581; Embase: 396), 244 studies were retrieved through Chinese databases (CNKI: 97; WanFang: 41; VIP: 56; SinoMed: 50), and eight studies were retrieved using Google Scholar. According to the inclusion criteria, 13 studies were included in this meta-analysis [4,5,29–32,35–41], including ten in English and three in Chinese language. Figure 1 shows the flow chart of the study eligibility process.

Seven high-quality studies (53.8%) and six medium-quality studies (46.2%) were included, and no study was considered of low quality (S2 Appendix).

### Characteristics of included studies

In the 13 studies, 107 participants with asymptomatic brucellosis were included. The majority of the cases were men and were distributed across all age groups. The follow-up duration ranged from 0.5 to 18 months. Multiple methods and criteria were used for serological diagnosis of brucellosis, but all studies employed SAT. The study regions included Asia (*n* = 10), Europe (*n* = 1), and North America (*n* = 1). The study population included high-risk occupational groups (shepherds, farmers, livestock, and veterinarians), students, and family members (specifically family members or neighbours with confirmed brucellosis). The specific characteristics and follow-up outcomes are shown in Tables 1 and 2, respectively.

**Table 1.** Characteristics of the included studies.

Included studies	Country	Investigation time	Age	Male (%)	Occupation	Main animals involved	Serological tests
Hu et al. (2021)	China	Nov, 2012 – May, 2014	median 34	60.0	Occupational population, Student	Sheep	SAT ≥ 1:100
Wu et al. (2019)	China	Jun – Aug, 2017	23–55	88.8	Occupational population	Cattle	SAT ≥ 1:100
Chen et al. (2019)	China	Apr, 2018 – Jan, 2019	1–62	53.3	Occupational population	Sheep	SAT ≥ 1:100
Mangalgi et al. (2016)	India	Oct – Nov, 2012	3–74	86.5	Occupational population	Livestock	SAT ≥ 1/160 and 2ME ≥1/80
Mangalgi et al. (2015)	India	NA*	1–74	60.0	Occupational population	Cattle, sheep and goat	SAT ≥ 1/160 and 2ME ≥1/80
Aghaali et al. (2015)	Iran	Jan – Dec, 2013	7–12	51.0	Student	Livestock	SAT ≥ 1/80 and 2ME ≥ 1/40 / Coombs ≥1/80
Ismayilova et al. (2013)	Azerbaijan	Aug, 2009	20–43	100.0	Family member**	Cattle, sheep and goat	SAT ≥ 1:200
Tabak et al. (2008)	Turkey	NA	4–55	50.0	Family member	Cattle	SAT ≥ 1:160
Zhang et al. (2008)	China	Three months of 2007	mean 38	100.0	Occupational population	Cattle	SAT ≥ 1:100
Alsubaie et al. (2005)	Saudi Arabia	Jan, 2001 – Feb, 2002	1-80, median 17	47.2	Family member	camel, sheep and goat	SAT ≥ 1:160
Almuneef et al. (2004)	Saudi Arabia	May, 2000 – Oct, 2001	2-67, mean 27	56.4	Family member	camel, sheep and goat	SAT ≥ 1:160
Young et al. (1991)	America	NA	47	100.0	Occupational population	Livestock	SAT/2ME ≥ 1:160***
Abramson et al. (1991)	Israel	Aug, 1988 – Aug, 1989	7–17	38.0	Family member	sheep and goat	SAT/2ME ≥ 1:160

*Not available. **Family members of the indexed cases (symptomatic brucellosis).

***SAT: Serum Agglutination Test; 2ME:2-mercaptoethanol.

**Table 2.** Follow-up outcomes and quality assessment of the included studies.

Included studies	Follow-up time (months)	Sample size	Follow-up outcomes			Quality assessment
			No. of appearing symptomatic	No. of maintaining asymptomatic	No. of decreased SAT titre	
Hu et al. (2021)	18	15	7	6	2	High
Wu et al. (2019)	2	6	3	NA*	NA	Medium
Chen et al. (2019)	5,6,9	4	0	NA	NA	Medium
Mangalgi et al. (2016)	1	13	0	NA	NA	Medium
Mangalgi et al. (2015)	1	6	0	NA	NA	Medium
Aghaali et al. (2015)	12	8	6	NA	NA	High
Ismayilova et al. (2013)	0.5-0.7	3	0	2	1	High
Tabak et al. (2008)	6–12	12	0	NA	NA	Medium
Zhang et al. (2008)	3	12	NA	NA	8	Medium
Alsubaie et al. (2005)	6–13	11	4	NA	NA	High
Almuneef et al. (2004)	6	11	2	NA	NA	High
Young et al. (1991)	17	1	0	1	0	High
Abramson et al. (1991)	12	5	1	1	3	High

*Not available.

### Meta-analysis

**Figure 2.**
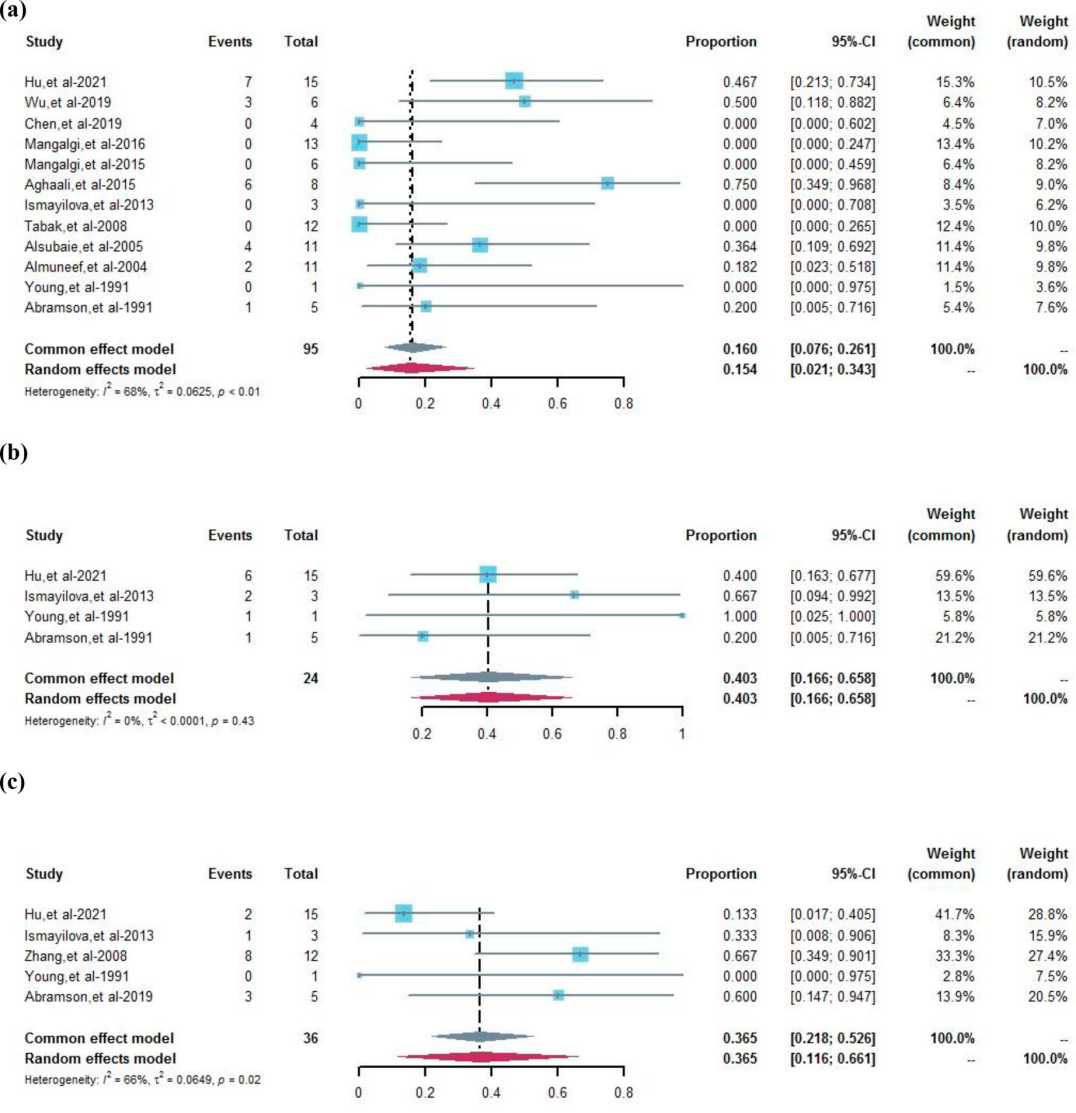
Forest plots of the pooled prevalence of follow-up outcomes. (a) Pooled prevalence of appearing symptomatic, weighted by random effects analysis. (b) Pooled prevalence of maintaining asymptomatic, weighted by fixed effects analysis. (c) Pooled prevalence of decreased SAT titre, weighted by random effects analysis. CI = confidence interval.

In the meta-analysis, 12 studies reported the follow-up outcome of appearing symptomatic. Due to the high heterogeneity among these studies (*I*^2 ^= 68%, *P *< 0.01), the random effects model was used for evaluating the pooled prevalence of appearing symptomatic was 15.4% (95%CI 2.1%–34.3%) (Figure 2(a)). Four studies reported follow-up outcomes of asymptomatic patients. We observed no heterogeneity among the studies (*I*^2 ^= 0%, *P *= 0.43); therefore, the pooled prevalence of asymptomatic status was 40.3% (95%CI 16.6%–65.8%) in the fixed effect model (Figure 2(b)). The pooled prevalence of decreased SAT titre in the five studies using random effects models (36.5%, 95%CI 11.6%–66.1%) with high heterogeneity (*I*^2 ^= 66%, *P *= 0.02) (Figure 2(c)).

### Results of publication bias and sensitivity analyses

No obvious asymmetry was found in the funnel plots of follow-up outcomes, and Egger’s tests were used to assess publication bias (appearing symptomatic, *P *= 0.8115; maintaining asymptomatic, *P *= 0.3735; decreased SAT titre, *P *= 0.8543). No publication bias was observed when using these indicators. In the sensitivity analyses, the estimated pooled prevalence of the follow-up outcomes was generally consistent, and the results were robust and reliable, except for decreased SAT titre (S4 Appendix).

### Results of subgroup analysis

In terms of the appearing symptomatic, the investigation-time subgroup showed a significantly higher pooled incidence (22.0%) in the last decade (2010–2021) than in the 1990–2009 period (8.4%). For regions, the pooled prevalence of appearing symptomatic was higher in Asia (20.9%), whereas this outcome was not observed in Europe and America, suggesting differences across regions. Only two studies from Europe and America were included, and their results should be interpreted with caution. Regarding population distribution, the highest pooled prevalence was in students (46.6%), with similar results for the occupational population and family members (11.9% and 11.7%, respectively). Regarding the main animals to which the study population was exposed, we found a pooled prevalence of appearing symptomatic, of 26.9% for camels, sheep, and goats, 23.9% for goats and sheep, and 15.3% for only cattle. In contrast, exposure to cattle, goats, and sheep did not result in this outcome (Table 3).

**Table 3.** Subgroup analysis of follow-up outcomes of appearing symptomatic.

Subgroups		No. of studies	Proportion (95% CI)	*I^2^*	*P*-value
Investigation time					
	1990–2009	6	0.084 (0.000, 0.226)	31%	0.21
	2010–2021	6	0.220 (0.006, 0.552)	81%	<0.01
Region					
	Asia	10	0.209 (0.054, 0.410)	68%	<0.01
	Europe & America	2	0.000 (0.000, 0.054)	0%	0.56
Population					
	Occupational population	6	0.119 (0.000, 0.466)	75%	<0.01
	Family member	5	0.117 (0.021, 0.254)	45%	0.13
	Student	2	0.466 (0.006, 0.969)	77%	0.04
Main animals					
	Sheep and/or goat	3	0.239 (0.010, 0.578)	51%	0.13
	Cattle	2	0.153 (0.000, 0.810)	86%	<0.01
	Cattle, sheep and goat	2	0.000 (0.000, 0.200)	0%	0.84
	Camel, sheep and goat	2	0.269 (0.094, 0.483)	0%	0.37
	Livestock	3	0.171 (0.000, 0.894)	88%	<0.01
Follow-up time					
	<6months	10	0.115 (0.003, 0.302)	70%	<0.01
	6–12months	6	0.264 (0.045, 0.552)	74%	<0.01
	6–18months	4	0.476 (0.255, 0.701)	31%	0.23
SAT					
	≥1:80/≥1:100	4	0.434 (0.144, 0.746)	59%	0.06
	≥1:160/≥1:200	8	0.036 (0.000, 0.130)	37%	0.14
Quality assessment					
	High	7	0.341 (0.195, 0.499)	38%	0.14
	Medium	5	0.028 (0.000, 0.206)	55%	0.06

Our results showed that the pooled prevalence of appearing symptomatic with follow-up times of < 6 months, 6–12 months, and 12–18 months was 11.5%, 26.4%, and 47.6%, respectively. The pooled prevalence of appearing symptomatic might have been higher with a longer follow-up period. The analysis showed that the pooled prevalence of appearing symptomatic was 43.4% based on the SAT titre ≥1:80/ ≥ 1:100, which was significantly higher than that based on the SAT titre ≥1:160/ ≥ 1:200 (3.6%). We also found that heterogeneity between the studies was relatively low (*I*^2 ^= 59% and 37%, respectively), and the SAT diagnostic criteria might be the source of heterogeneity. In addition, subgroup analysis of the quality of literature showed that a higher pooled prevalence of appearing symptomatic outcomes occurred in high-quality studies (34.1%) than in medium-quality studies (2.8%). This might also be a source of heterogeneity according to the heterogeneity analysis of the high and medium quality studies (*I*^2 ^= 38% and 55%, respectively). The details are listed in Table 3.

## Discussion

This study represents the first systematic evaluation of follow-up outcomes of asymptomatic brucellosis. Our analysis found that the highest pooled prevalence was maintaining asymptomatic (40.3%), the lowest was appearing symptomatic (15.4%), and decreased SAT titre was 36.5% during the 0.5–18 months follow-up period. These results suggest that a high percentage of SAT titres decreased or remained at a certain level, even if asymptomatic individuals were not treated. Brucellosis antibodies in the body are less durable, which is consistent with previous reports [31,38].

*Brucella* spp. are facultative intracellular bacteria that can limit or avoid the host immune response and replicate within the host cells [42]. Once infected, it induces long-lasting infections that develop into chronic or recurrent diseases, instead of complete eradication of the bacteria from the body. Therefore, the appearance of symptoms is likely to attract attention and concern. In our study, although there was a relatively small proportion of appearing symptomatic cases, the high proportion of potential cases and risks of morbidity associated with asymptomatic brucellosis cannot be ignored. We found that follow-up time was an important factor influencing follow-up outcomes. In the subgroup analysis of follow-up outcomes of appearing symptomatic, the pooled prevalence also increased to some extent with a longer follow-up. Consequently, it is reasonable to assume that, for longer follow-up periods, the pooled prevalence of appearing symptomatic will exceed the current levels. When we evaluated high-quality literature separately, we also found that we might have grossly underestimated the actual pooled prevalence of appearing symptomatic. The pooled prevalence of appearing symptomatic was significantly higher (34.1%) than that reported in medium-quality studies (2.8%). Considering the effect of follow-up time, it is safe to suggest that the prevalence of appearing symptomatic may be much higher than that reported by the current studies.

Our study showed that the pooled prevalence of appearing symptomatic in the last decade was significantly higher than that in the period 1990–2009. There are two possible reasons for this observation. One is that with the ever-improving quality of life, the manner and frequency of exposure to contaminated animals or dairy products have increased dramatically, and repeated infections increase the likelihood of appearing symptomatic. The other is the improvement in medical care, which allows early symptoms to be diagnosed and monitored in a timely manner [43]. The situation is becoming more and more critical for the emergence of symptoms in asymptomatic patients with brucellosis. Notably, the *I*^2^ test suggested that the quality of the literature may be an important source of heterogeneity in appearing symptomatic group.

Our study also showed that the majority of participants were from Asia and only 12% of the participants were from Europe and America. This is generally consistent with the global distribution of brucellosis in Asian and African regions [44]. The lack of reports from Africa may be due to poor medical services, making it difficult to turn the attention to asymptomatic groups. In addition, we found that cases were almost evenly distributed across age groups, which could be explained by the fact that family members and occupational groups screened for brucellosis shared the same source of infection and similar risk factors [45]. Previous studies have concluded that asymptomatic brucellosis may be related to the epidemic intensity of animal brucellosis, contact patterns, frequency of contact with animals, and personal immunity [16,40]. Similarly, most cases were in brucellosis endemic areas, and all had a history of frequent exposure to animals and their products, as shown in our study. Most asymptomatic patients were men because they are more likely to work in high-exposure occupations such as veterinarians, slaughter workers, or herders [3]. Asymptomatic brucellosis has both occupational and familial clusters.

Students are worthy of attention in terms of population distribution. The pooled prevalence of appearing symptomatic was 46.6%, which was higher than that of the high-risk occupational population (11.9%). This may be related to the relatively young age of students, their developing and weak immune system [46]. In contrast, certain occupational groups, such as herders, farmers, veterinarians, and butchers that are at a higher risk of infection for a long time, have higher levels of antibodies to *brucella* and are more tolerant. Interestingly, occupational groups had the same pooled prevalence as family members. Family members were screened based on the index of confirmed cases [4,30,38]. Thus, they had a similar exposure history to the index cases, a higher risk of transmission, and a largely convergent source and course of infection [37]. In summary, it is necessary to actively screen and monitor the family members of brucellosis patients and high-exposure occupational groups in endemic areas. With this screening approach, unrecognized cases, especially students, can be identified earlier in the course of the disease, and early diagnosis and intervention can reduce the occurrence of chronic diseases and complications [29].

Therefore, the outcome of appearing symptomatic is increasing, and the possibility of serious consequences also increasing by “waiting” for treatment. Nevertheless, the reality is that we seem unprepared for this challenge; high-risk regions and occupational populations are not immune. In general, early diagnosis and intervention are more conducive to the early cure of diseases. However, regarding asymptomatic brucellosis, probably owing to concerns about drug side effects, overmedication, and underestimation of the serious outcomes of asymptomatic infections, there has been no clear means of intervention (or treatment) [10], causing considerable confusion in the patients. Although patients are concerned about possible serious chronic damage to the joints, nervous system, and reproductive system [47], doctors are unable to offer precise treatment advice and can only wait for the appearance of related symptoms before administering medicine. It must be noted that, based on the limited information we currently have, it is difficult to determine whether asymptomatic brucellosis should be equated with confirmed cases and provide adequate or preventative medication. However, we still hope to provide a clinical reference for diagnosis and treatment, as well as to give patients some judgment of the outcome and reduce confusion. Finally, we offer the following suggestions. First, the active screening and surveillance of occupational groups and their family members should be strengthened. After screening for cases (including asymptomatic brucellosis), the source of infection should be identified as early as possible to avoid re-exposure and strengthen their protection. Second, attention should be paid to the prevalence of brucellosis seropositivity in student groups in endemic areas, and if necessary, preventive medication can be used for those with high titre. Third, education and public awareness of asymptomatic brucellosis should be strengthened reduce the contact with infected animals and consumption of unpasteurized animal products.

However, our study has several limitations. First, Egger’s tests showed no significant publication bias among the included studies, but the small number of studies and sample size may limit the strength of the study. Second, there was still some heterogeneity in the meta-analysis, although we have explained some of the possible sources of heterogeneity.

Due to the limitations of the study design and sample size, we cannot provide definitive conclusions on whether asymptomatic brucellosis patients should be treated. In conclusion, further prospective long-term follow-up studies with larger sample sizes are needed to provide more evidence.

## Supplementary Material

Supplemental Material

## Data Availability

All data generated or analyzed during this study are included in this published article and its supplementary files.
