# Supplementary material for: Follow-up outcomes of asymptomatic brucellosis: a systematic review and meta-analysis
Source: Emerg Microbes Infect. 2023 Mar 13;12(1):2185464. doi: 10.1080/22221751.2023.2185464 (PMC10013368; doi:10.1080/22221751.2023.2185464)
Supplement: Supplemental Material [file TEMI_A_2185464_SM7614.zip › S1 Appendix.Studies search strategies in the meta-analysis.docx]

**S1 Appendix. Studies search strategies in the meta-analysis**

**1.English Database**

**PubMed：**

(Brucellosis[Mesh] OR Brucellosis[Title/Abstract] OR Brucelloses[Title/Abstract] OR "Malta Fever"[Title/Abstract] OR "Undulant Fever"[Title/Abstract] OR "Brucella Infection*" [Title/Abstract] OR "Rock Fever"[Title/Abstract] OR "Gibraltar Fever"[Title/Abstract]) AND (Follow-Up Studies[Mesh] OR Follow-Up[Title/Abstract] OR Followup[Title/Abstract] OR Asymptomatic Diseases[Mesh] OR Asymptomatic Infections[Mesh] OR Asymptomatic[All Fields] OR Subclinical[All Fields] OR Presymptomatic[All Fields] OR Inapparent[All Fields] OR Outcome[Title/Abstract])

**Web of Science:**

((TS=(Brucellosis OR Brucelloses OR Malta Fever OR Undulant Fever) OR TI=(Brucellosis OR Brucelloses OR Malta Fever OR Undulant Fever OR Brucella Infection* OR Rock Fever OR Gibraltar Fever) OR AB=(Brucellosis OR Malta Fever OR Undulant Fever OR Brucella Infection* OR Rock Fever OR Gibraltar Fever)) AND ((TS=(Asymptomatic Diseases OR Asymptomatic Infections OR Follow-Up Studies OR Asymptomatic Infections) OR TI=(asymptomatic OR Subclinical OR Presymptomatic OR Inapparent OR Follow-Up Studies OR Follow-Up OR Followup OR Outcome) OR AB=(asymptomatic OR Subclinical OR Presymptomatic OR Inapparent OR Follow-Up OR Followup OR Outcome)

**Scopus:**

((TITLE-ABS-KEY (brucellosis OR brucelloses OR malta fever OR undulant fever OR brucella infection* OR rock fever OR gibraltar fever)) AND (ALL ( asymptomatic OR presymptomatic OR inapparent) OR TITLE-ABS-KEY(asymptomatic diseases OR asymptomatic infection* OR follow-up OR followup OR outcome)

**Embase**

('brucellosis'/exp OR brucellosis:ab,ti OR brucelloses:ab,ti OR 'malta fever':ab,ti OR 'undulant fever':ab,ti OR 'brucella infections':ab,ti OR 'rock fever':ab,ti OR 'gibraltar fever':ab,ti) AND ('asymptomatic diseases'/exp OR 'asymptomatic infections'/exp OR asymptomatic OR subclinical OR presymptomatic OR inapparent OR 'follow-up studies'/exp OR 'follow up':ab,ti OR followup:ab,ti OR outcome:ab,ti)

**2.Chinese Database**

**China National Knowledge Infrastructure (CNKI):**

((Brucellosis[SU] OR Brucellosis[KTA] OR Brucella[SU] OR Brucella[KTA] OR "Rock Fever"[SU] OR "Rock Fever"[KTA]) AND (Asymptomatic[FT] OR Subclinical[FT] OR Presymptomatic[FT] OR Inapparent[FT]) AND (Follow-Up[SU] OR Follow-up[KTA] OR Outcome[SU] OR Outcome[KTA] OR Family[KTA] OR Screen[KTA] OR Occupation[KTA] )

**Wan Fang:**

Theme:(Brucellosis OR Brucella OR "Malta Fever" OR "Rock Fever") AND (All:(Asymptomatic OR Subclinical OR Presymptomatic OR Inapparent)) AND Title/Keyword/Abstract:(Follow-Up OR Family OR Screen OR Occupation OR Outcome )

**VIP Chinese Journal Databases:**

(M=(Brucellosis OR Brucella OR Malta Fever OR Rock Fever ) OR R= (Brucellosis OR Brucella OR Malta Fever OR Rock Fever)) AND (U=(Asymptomatic OR Subclinical OR Presymptomatic OR Inapparent )) AND (M=(Asymptomatic OR Subclinical OR Presymptomatic OR Inapparent) OR R=(Follow-Up OR Family OR Screen OR Occupation OR Outcome))

**SinoMed:**

(("Brucellosis"[Title/Abstract/Keyword] OR "Brucella"[Title/Abstract/Keyword] OR "Rock Fever"[Title/Abstract/Keyword]) AND (Asymptomatic[ALL] OR Subclinical[ALL] OR Presymptomatic[ALL] OR Inapparent[ALL])) AND (Follow-Up[Title/Abstract/Keyword] OR Family[Title/Abstract/Keyword] OR Screen[Title/Abstract/Keyword] OR Occupation[Title/ Abstract/Keyword] OR Outcome[Title/Abstract/Keyword])
