# Supplementary material for: Follow-up outcomes of asymptomatic brucellosis: a systematic review and meta-analysis
Source: Emerg Microbes Infect. 2023 Mar 13;12(1):2185464. doi: 10.1080/22221751.2023.2185464 (PMC10013368; doi:10.1080/22221751.2023.2185464)
Supplement: Supplemental Material [file TEMI_A_2185464_SM7614.zip › S2 Appendix. Quality assessment.docx]

**S2 Appendix. Quality assessment**

The US Agency for Healthcare Research and Quality (AHRQ) recommended criteria for evaluating cross-sectional studies including the following 11 items. Answer with "yes", "no" and "unclear" respectively, choose "yes" for 1 point, "unclear" or "no" for 0 point. The higher the score, the higher the quality of the literature.

①Define the source of information (survey, record review);

②List inclusion and exclusion criteria for exposed and unexposed subjects (cases and controls) or refer to previous publications;

③Indicate time period used for identifying patients;

④Indicate whether or not subjects were consecutive if not population-based;

⑤Indicate if evaluators of subjective components of study were masked to other aspects of the status of the participants;

⑥Describe any assessments undertaken for quality assurance purposes (e.g. test/retest of primary outcome measurements);

⑦Explain any patient exclusions from analysis;

⑧Describe how confounding was assessed and/or controlled;

⑨If applicable, explain how missing data were handled in the analysis;

⑩Summarize patient response rates and completeness of data collection;

⑪Clarify what follow-up, if any, was expected and the percentage of patients for which incomplete data or follow-up was obtained.

**The quality assessment scores of the included literature in this study:**

| **No.** | **Included studies** | **①** | **②** | **③** | **④** | **⑤** | **⑥** | **⑦** | **⑧** | **⑨** | **⑩** | **⑪** | **Total** | **Quality** |
| --- | --- | --- | --- | --- | --- | --- | --- | --- | --- | --- | --- | --- | --- | --- |
| 1 | Hu, et al.(2021) | 1 | 1 | 1 | 1 | 1 | 1 | 1 | 1 | 1 | 1 | 1 | 11 | High |
| 2 | Wu, et al.(2019) | 1 | 1 | 1 | 0 | 1 | 1 | 1 | 0 | 0 | 0 | 0 | 6 | Medium |
| 3 | Chen, et al.(2019) | 1 | 1 | 1 | 0 | 1 | 0 | 1 | 0 | 0 | 0 | 0 | 5 | Medium |
| 4 | Mangalgi, et al.(2016) | 1 | 1 | 1 | 0 | 1 | 1 | 1 | 0 | 0 | 0 | 0 | 6 | Medium |
| 5 | Mangalgi, et al.(2015) | 1 | 1 | 0 | 0 | 1 | 1 | 1 | 1 | 0 | 0 | 0 | 6 | Medium |
| 6 | Aghaali, et al.(2015) | 1 | 1 | 1 | 0 | 1 | 1 | 1 | 1 | 0 | 1 | 0 | 8 | High |
| 7 | Ismayilova, et al.(2013) | 1 | 1 | 1 | 1 | 1 | 1 | 1 | 0 | 1 | 1 | 1 | 10 | High |
| 8 | Tabak, et al.(2008) | 1 | 1 | 0 | 0 | 1 | 0 | 1 | 0 | 0 | 0 | 0 | 4 | Medium |
| 9 | Zhang, et al.(2008) | 1 | 1 | 1 | 0 | 1 | 1 | 1 | 1 | 0 | 0 | 0 | 7 | Medium |
| 10 | Alsubaie, et al.(2005) | 1 | 1 | 1 | 0 | 1 | 1 | 1 | 0 | 1 | 1 | 0 | 8 | High |
| 11 | Almuneef, et al.(2004) | 1 | 1 | 1 | 0 | 1 | 1 | 1 | 1 | 1 | 0 | 0 | 8 | High |
| 12 | Young, et al.(1991) | 0 | 1 | 0 | 1 | 1 | 1 | 1 | 1 | 1 | 1 | 1 | 9 | High |
| 13 | Abramson, et al.(1991) | 1 | 1 | 1 | 1 | 1 | 1 | 1 | 0 | 1 | 0 | 1 | 9 | High |
