# Supplementary material for: Follow-up outcomes of asymptomatic brucellosis: a systematic review and meta-analysis
Source: Emerg Microbes Infect. 2023 Mar 13;12(1):2185464. doi: 10.1080/22221751.2023.2185464 (PMC10013368; doi:10.1080/22221751.2023.2185464)
Supplement: Supplemental Material [file TEMI_A_2185464_SM7614.zip › S4 Appendix. Funnel plots and sensitivity analyses.docx]

**S4 Appendix. Funnel plots and sensitivity analyses**

**
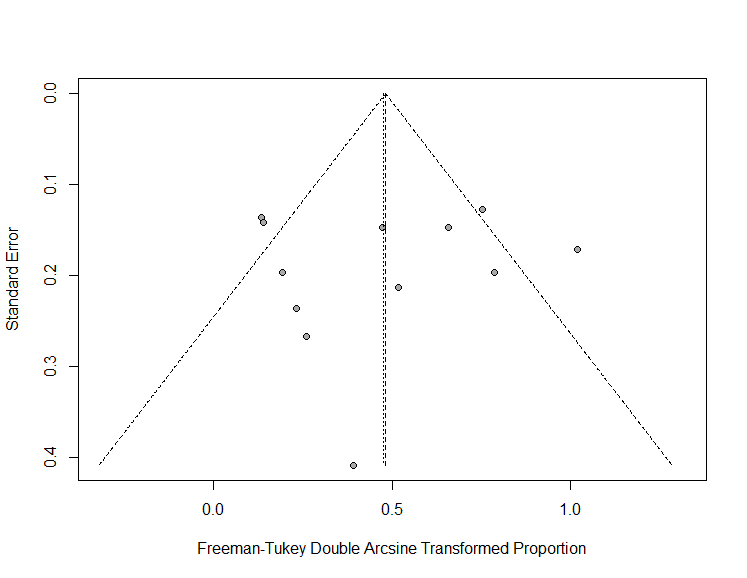
**

**Figure 1A. Funnel plot of appearing symptomatic**

**
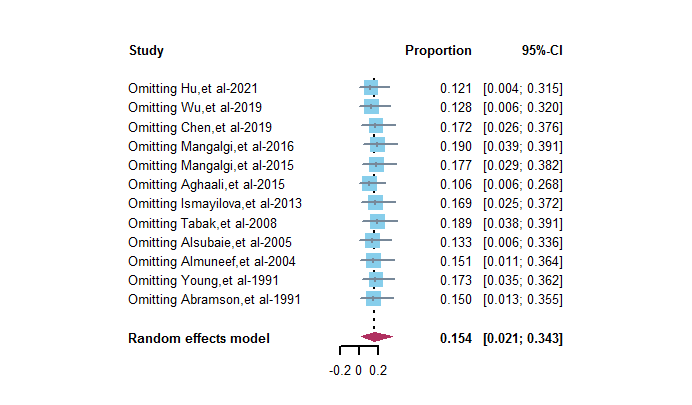
**

**Figure 1B. Sensitivity analyses of appearing symptomatic**

**
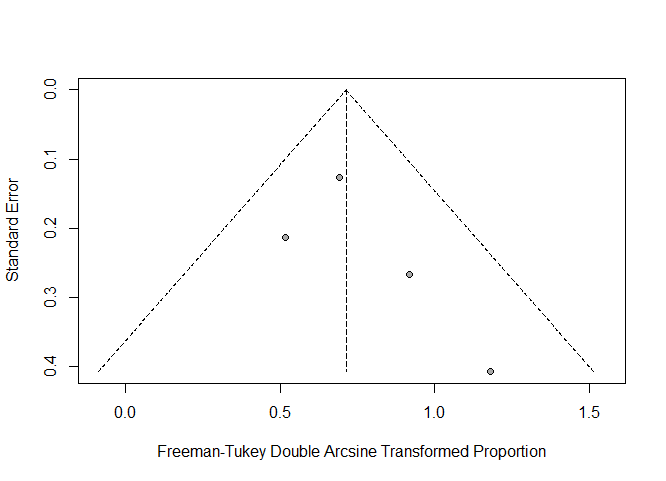
**

**Figure 2A. Funnel plot of maintaining asymptomatic**

**
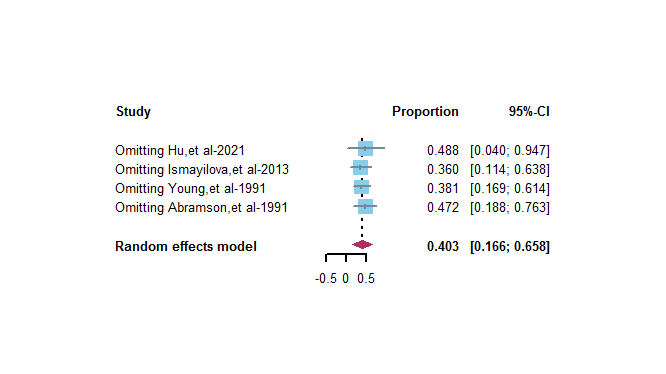
**

**Figure 2B. Sensitivity analyses of maintaining asymptomatic**

**
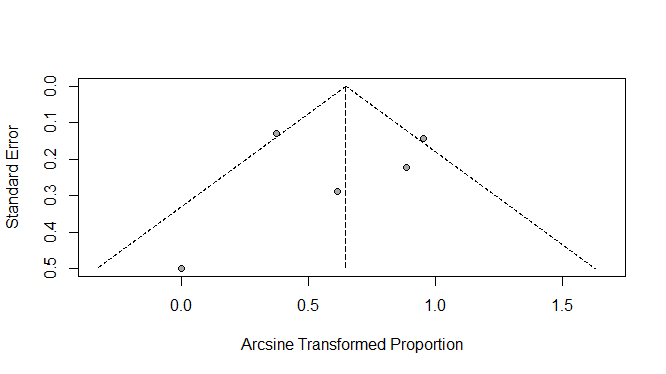
**

**Figure 3A. Funnel plot of decreased SAT titer**

**
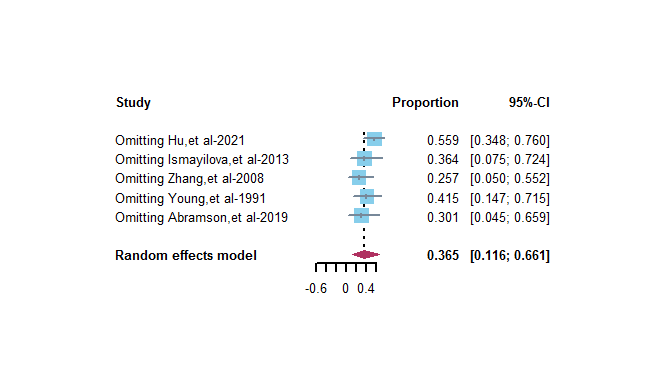
**

**Figure 3B. Sensitivity analyses of decreased SAT titer**
